# Photonic advantage of optical encoders

**DOI:** 10.1515/nanoph-2023-0579

**Published:** 2023-11-16

**Authors:** Luocheng Huang, Quentin A. A. Tanguy, Johannes E. Fröch, Saswata Mukherjee, Karl F. Böhringer, Arka Majumdar

**Affiliations:** Electrical and Computer Engineering, University of Washington, Seattle, WA, 98195, USA; Physics Department, University of Washington, Seattle, WA, 98195, USA; Department of Bioengineering, Institute for Nano-Engineered Systems, University of Washington, Seattle, WA, 98195, USA; Physics Department, Electrical and Computer Engineering, University of Washington, Seattle, WA, 98195, USA

**Keywords:** optical encoder, metasurface, optical neural network

## Abstract

Light’s ability to perform massive linear operations in parallel has recently inspired numerous demonstrations of optics-assisted artificial neural networks (ANN). However, a clear system-level advantage of optics over purely digital ANN has not yet been established. While linear operations can indeed be optically performed very efficiently, the lack of nonlinearity and signal regeneration require high-power, low-latency signal transduction between optics and electronics. Additionally, a large power is needed for lasers and photodetectors, which are often neglected in the calculation of the total energy consumption. Here, instead of mapping traditional digital operations to optics, we co-designed a hybrid optical-digital ANN, that operates on incoherent light, and is thus amenable to operations under ambient light. Keeping the latency and power constant between a purely digital ANN and a hybrid optical-digital ANN, we identified a low-power/latency regime, where an optical encoder provides higher classification accuracy than a purely digital ANN. We estimate our optical encoder enables ∼10 kHz rate operation of a hybrid ANN with a power of only 23 mW. However, in that regime, the overall classification accuracy is lower than what is achievable with higher power and latency. Our results indicate that optics can be advantageous over digital ANN in applications, where the overall performance of the ANN can be relaxed to prioritize lower power and latency.

## 1 Introduction

Over the last decade, the fields of artificial intelligence (AI) and deep learning have experienced accelerated progress, revealing the potential and capabilities of artificial neural networks (ANN) for a variety of applications, with recent demonstrations even advancing to the public spotlight in the form of chat software and artistic rendering programs. Their recent success can be traced back to major breakthroughs, both in terms of computational algorithms and digital hardware such as graphics processing units (GPU) [1]. While impressive, the scaling of power and latency of digital implementations of deep learning turned out to be unfavorable with the size of the ANN. This poses a serious limitation for further scaling of ANNs [2, 3] and applicability to low-power, real-time problems.

Light may be the answer to this scaling challenge, thanks to its inherent parallelism, speed, and analog nature, thus providing an attractive alternative to electronic implementations to build energy efficient and fast ANNs. This has been recognized early on and several experiments reported optical ANNs already back in the 1990s [4, 5]. Unfortunately, progress stalled due to technological and fundamental reasons, which can be broadly classified into intrinsic and extrinsic problems. Intrinsic problems with optics had been the large size and poor tolerance to misalignment of optical components; limited space bandwidth product of spatial light modulators; and lack of nonlinear activation. The extrinsic problems originated from poor understanding of AI algorithms and adaptive learning, as well as the meteoric rise of electronic computing systems.

Given the current limitations of electronic hardware and our increased understanding of AI, the extrinsic problems are somewhat alleviated. In parallel, the advancement of nano-fabrication facilities, and the availability of sophisticated electromagnetic simulators have led to the high-volume manufacturing of multi-functional nano-optics, such as flat meta-optics [6, 7] and integrated photonic devices [8]. Emerging material systems coupled with these nano-optical structures enable monolithic photonic integrated circuits (PIC) analogous to electronic ICs [9]. These innovations in nanophotonics and AI, combined with severe limitations of digital implementation of ANNs have generated strong interest in recent years in recreating optics assisted ANNs [10–17].

However, thus far, none of the reported works have demonstrated a clear advantage of optics over digital ANNs for inference. Most implementations have only shown the substitution of a small linear part with an optical counterpart [18], while the rest was kept in the digital electronics. Although there is a clear advantage of optics for implementing a small sub-system, often the linear part, the power and latency in a complete ANN include the transduction of the signal between optical and electronic domains [19], i.e. the detector readout power, spatial light modulator power and laser power, many of which are often neglected. In fact, an analysis considering these energy costs shows that implementing only one convolutional layer in optics does not provide any advantage, unless the input has a very large dimension [19]. However, for many applications, such large dimensions of the image provide only a marginal increase in ANN classification accuracy. There are several recent works that also implemented nonlinearity in the optical domain using thermal atoms [16] and image intensifiers [20]. These approaches, however, also consume a large amount of power. Additionally, a large body of works demonstrated classification for extremely simple “toy” problems, for which no digital benchmark exists [13, 14]. Comparing the power and latency of an application specific optical ANN to a GPU (optimized for universal operations) is unfair. There are many ways to drastically reduce the power and latency of a digital ANN, including replacing matrix multiplication with XNOR operations [21]. Many pruning algorithms also exist to reduce the number of computations needed for inference. As such, there has been no clear demonstration where an optics-assisted ANN shows an advantage over a purely digital framework optimized for solving a specific problem. The current approaches generally focus on power and speed benefit form inclusion of optics to achieve similar classification accuracy. However, it is impossible to exactly define the computational complexity of an ANN; hence the exact calculation of power and latency in the digital part is dependent on both training and technology.

**Figure 1:**
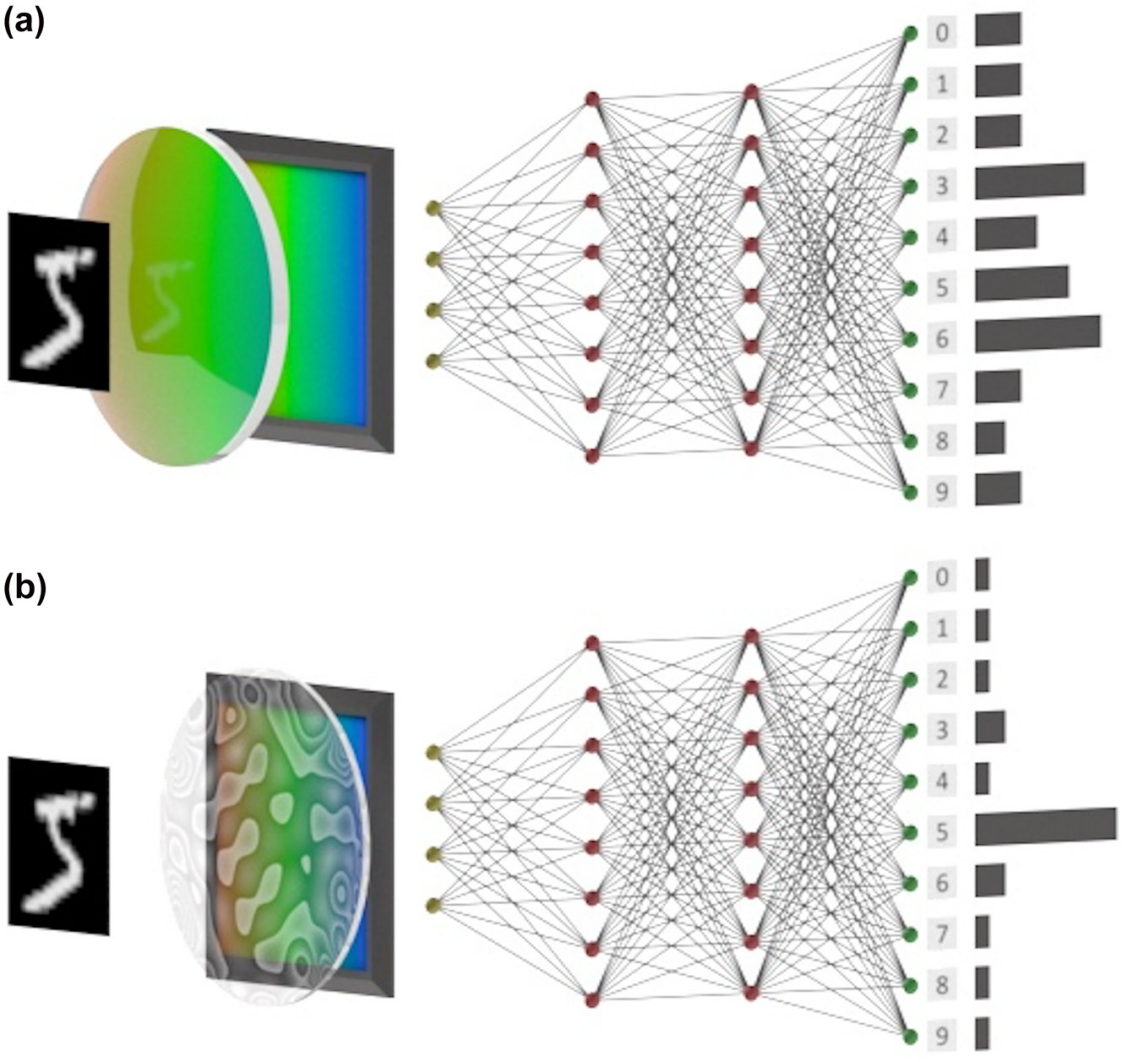
Schematic of the optical encoder and pure digital neural network. (a) Purely digital ANNs operate on captured images using a lensed sensor. (b) Instead of using a lens, a designed optics can perform additional linear operations on the captured data. In both cases, the power and the latency of the sensor are the same. Using the digital computational backend with the same resources (number of layers and neurons), we ensure the same power and latency, both of which monotonically scale with the dimensionality of the input data (here termed as *N*) to the digital backend.

Here, we develop a framework to exactly compare the inference performance of a pure digital ANN against a hybrid optical-digital ANN. In both ANNs, we ensure the same power and latency, and thus by comparing the classification accuracy, we can clearly assess the relative advantage. Figure 1 shows a schematic of the two cases: the pure digital and the hybrid optical-digital. We encode the input in incoherent light, as the optical frontend of the ANN can work with ambient light without incurring any additional energy consumption. In a pure digital case, a lens-based sensor captures an image of an object under incoherent light, and then the image is transferred to a digital ANN. For the hybrid case, we use an engineered optic – namely the optical encoder, instead of a lens, that captures the image in a different basis and sends the data to a digital backend. Instead of implementing a digital sub-system, such as convolutional operations in optics, we co-optimize the optical frontend (implemented via a sub-wavelength diffractive meta-optics), along with the digital backend using an “end-to-end” design framework (detail in the Supplementary Materials S1, S3) [22, 23]. The topology and resources (i.e., the same number of nodes, layers, and nonlinearities) used in the digital ANN are kept the same in both cases, though with different weights and biases. Thus, we ensure that the latency and power consumption in both cases remain identical. We note that the designed meta-optic essentially performs a convolutional operation, but with a significantly larger kernel size compared to standard convolutional neural networks. This can be justified by the fact that any image formation under incoherent illumination can be modelled as a convolution between the object and the incoherent point spread function (PSF), if the PSF is spatially uniform. While meta-optics does not strictly have a spatially invariant PSF, and such spatial variation is recently been exploited for convolution [24], this approximation has worked well for many other imaging applications [22, 25].

Here, we tested the classification accuracy for MNIST data sets for different values of *N*, which represent the binned size of the image captured in the sensor either via a lens or the optical encoder. As the latency and power increase with the input dimensionality *N* of the data sent to the digital ANN, we found that classification accuracy increases in both cases, and there is no advantage from an optical frontend for large *N*. However, for smaller *N*, where the system power and latency are also lower, we found an increase in validation accuracy (∼10 %) with a hybrid optical-digital ANN. We experimentally validated our theoretical model. Our work clearly demonstrates a photonic advantage for ANN inference, albeit such an advantage is observed when overall system performance is lower than the highest achievable performance.

## 2 Results

**Figure 2:**
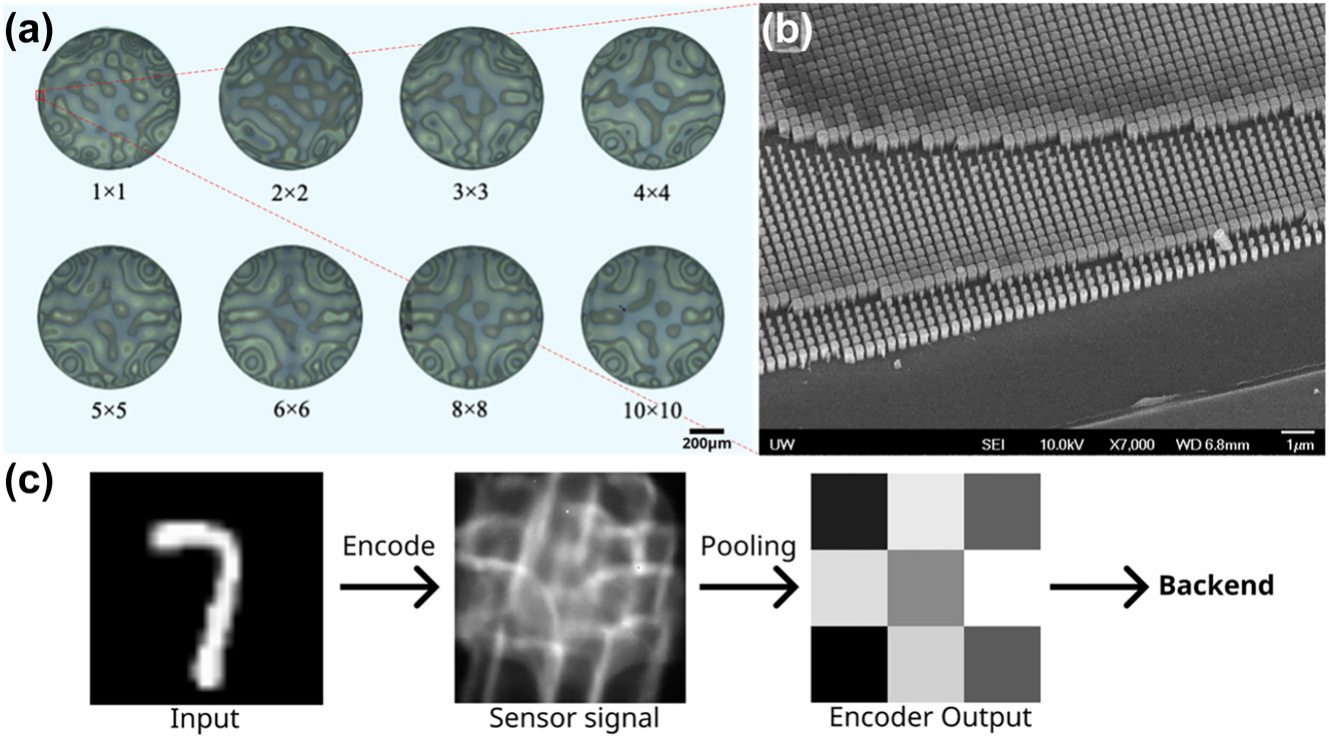
Fabrication and characterization of the meta-optical encoder: (a) Optical microscope images of the meta-optical encoders for different input sizes. (b) Scanning electron microscope (SEM) image of the optical encoder, region denoted by the red box on device 1 × 1. (c) The experimental input, sensor signal, and output of the meta-optical encoder.

**Figure 3:**
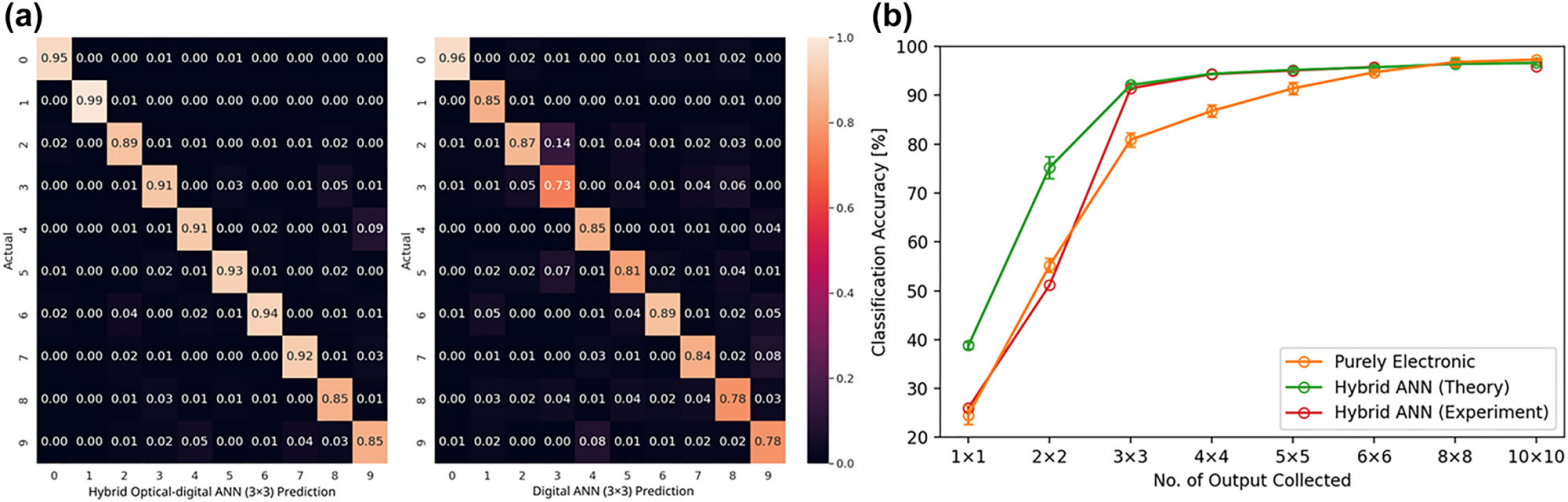
Performance comparison of the digital and hybrid ANN. (a) Confusion matrices comparing the experimental performances of the hybrid optical-digital against the pure digital ANNs for the case of *N* = 3 × 3. (b) Validation classification accuracies of the purely electronic and hybrid optical-electronic ANNs as a function of *N*, N being the number of output points being transferred to the computational backend. The error bar is shown to represent the range of one standard deviation.

Our digital backend consists of three fully connected layers: *N* × 256 (input), 256 × 256 (hidden) and 256 × 10 (output). The first two layers are each followed by a rectified linear unit (ReLU) nonlinearity and the output layer has a sigmoid nonlinearity. For the pure digital case, every image is converted to an N-pixel image by averaging the pixels. We chose 8 different *N* ranging from 1 to 100, to assess the performance of the system with increasing data input. We train the digital network by back-propagating the loss function defined by the cross-entropy between the output and the ground truth. In simulation, we obtained a validation classification accuracy of up to ∼98 % (detail in the Supplementary Materials S2). We note that, in prior works, to achieve a similar accuracy with the MNIST dataset, several layers were used [17], which we attribute to inefficient training. For the hybrid case, we model the optical frontend using a sub-wavelength diffractive meta-optics, although any freeform optical surface could suffice for implementation. The fabricated optical frontends with different output dimensionalities are shown in Figure 2(a). We train the meta-optics along with a digital backend with the same neural network topology (details in method), following an “end-to-end” design framework used before for imaging [20]. For training we assumed the light is incoherent but monochromatic. As expected, we observed an increase in classification accuracy with increasing *N*. We also found that for *N* > 8 × 8, the digital and hybrid ANN demonstrate identical classification accuracies. However, at a lower value of *N*, the classification accuracy of the hybrid ANN surpasses that of the digital ANN. Example classification confusion matrices are shown in Figure 3(a), comparing the experimental validation accuracies between a hybrid and a digital ANN with the same input size, *N* = 3 × 3. Theoretically, we observe an increase in classification accuracy by up to ∼20 % when an optical frontend is incorporated. A validation accuracy comparison chart can be seen on Figure 3(b). We note that even with a single data-point sent to the digital backend, we theoretically achieved higher classification accuracy with our optical frontend. This is because that single input can assume 256 different values for an 8 bit precision sensor, which can help with classification. We discovered that if we use a lower bit resolution instead of an 8 bit resolution in the output, the classification accuracy drastically declines for small *N*.

To validate the design, we fabricated the meta-optics (detail in the Supplementary S4) and measured their performance experimentally, where we projected images of the MNIST data set using an OLED display in green (detail in the Supplementary S5). The incoherent green light passes through the meta-optic, and we capture the data on the sensor with 8 bit precision. We then binned the captured image to create the N data-points that are passed to the digital backend. An experimental sample on Figure 2(c) shows the signal processing of the 3 × 3 encoder. Due to fabrication imperfections, and misalignments, we retrained the digital backend (keeping the same topology) using the captured data. Our experiment matches the theory very well for *N* ≥ 3 × 3. We note that the meta-optics optimized for *N* = 8 × 8 was damaged, and we could not collect data on that. At smaller *N*, the deviation from the theory is attributed to experimental noise. While a single point can provide more information to the digital backend, it is corrupted by the quantization noise, undermining the effect of the optical encoder and we obtained similar classification accuracy, as we would have expected from a pure digital backend. We have also verified this in simulation: by reducing the bit resolution and adding more quantization noise, the classification accuracy degrades more for *N* = 1 and *N* = 4.

## 3 Discussion

By employing an incoherent light source and a meta-optical frontend, we created a framework, enabling us to compare the performance of a digital ANN to an optics-assisted ANN in the same footing. While keeping the power and latency constant in both cases, we showed that optical encoding does provide more information to the digital backend, resulting in ∼10 % more classification accuracy in the experiment. We emphasize that to achieve >90 % classification accuracy for the hybrid case, it is only necessary to capture a 3 × 3 image, i.e., nine pixels on the sensor. In contrast, for the same image size, the classification accuracy of the pure electronic method remains at approximately 80 %. The power of the hybrid optical ANN can be estimated from the sensor readout power and the power utilized by the digital backend. The sensor readout power is directly proportional to the number of pixels. For a typical commercial camera, we estimate the sensor readout power for a 9-pixel image to be around 18 mW at a speed of approximately 10 kHz. Given *N* inputs, the backend needs to execute a total of approximately $\left(5\times 10^{5}+2\times 10^{3}\times N\right)$ multiply-and-accumulate (MAC) operations with 8 bit precision. In modern digital system, one MAC operation uses about ∼1 pJ [20], making the total energy for the digital backend *N* = 3 × 3 to be ∼500 nJ. Given our proposed network will be limited by the sensor readout time, we can estimate the backend power to be ∼5 mW. Thus, our reported hybrid ANN consumes ∼23 mW power for ∼10 kHz operating speed. This low energy originates from the fact that our optical operations are hard coded in an engineered optics. Additionally, by capturing only a few pixels we drastically reduce the sensor power. However, the price we pay is that unlike spatial light modulators, we cannot reconfigure the frontend. As such, this power should be considered as a lower limit of the sensor and compute operations for MNIST datasets. Another benefit of our hybrid network is its simplicity. We only need one meta-optic, which can be directly integrated into the sensor. Unlike 4f systems [26], or multiple meta-optics [15], use of a single meta-optic drastically reduces the size, weight, and packaging complexity of our encoder.

While our result is primarily applicable to the MNIST dataset, we believe that it indicates the conditions for which an optical frontend is beneficial to increase the performance of an ANN (more discussion in Supplementary S6). Without any constraints on latency and power, one can arbitrarily increase *N*, and always find a digital solution that is better than the hybrid option. One way to rationalize this is that any optical implementation can be modelled digitally and therefore without any constraint a digital solution can be found with accuracy in the same order of magnitude or higher than its optical counterpart. The higher classification accuracy of optics-assisted ANN in several reports is most likely a manifestation of poor training of the fully digital ANN. However, under the constraints of latency or power, we need to work with an intermediate value of *N*, where the optical frontend can provide a more efficient solution, albeit at overall lower accuracy.

## Supplementary Material

Supplementary Material Details
