# Supplementary material for: Photonic advantage of optical encoders
Source: Nanophotonics. 2023 Nov 16;13(7):1191–6. doi: 10.1515/nanoph-2023-0579 (PMC11501926; doi:10.1515/nanoph-2023-0579)
Supplement: Supplementary file 1 — Supplementary Material Details [file j_nanoph-2023-0579_suppl_001.docx]

**Supplementary Materials for**

**Photonic Advantage of Optical Encoders**

Authors: Luocheng Huang^1^, Quentin A. A. Tanguy^1^, Johannes E. Fröch^1,2^, Saswata Mukherjee^1^, Karl F. Böhringer^1,3,4^, Arka Majumdar^1,2, *^

**This PDF file includes:**

**S1. End-to-end design of the meta-optics and digital backend**

**S2. Design of Purely Digital neural network**

**S3. Design of the meta-optics**

**S4. Fabrication of the meta-optics**

**S5. Meta-optical encoder experimental details**

**S6. The demonstration of benefit of optics in the existing work**

**S1. End-to-end design of the meta-optics and digital backend: the hybrid optical-digital ANN**

**
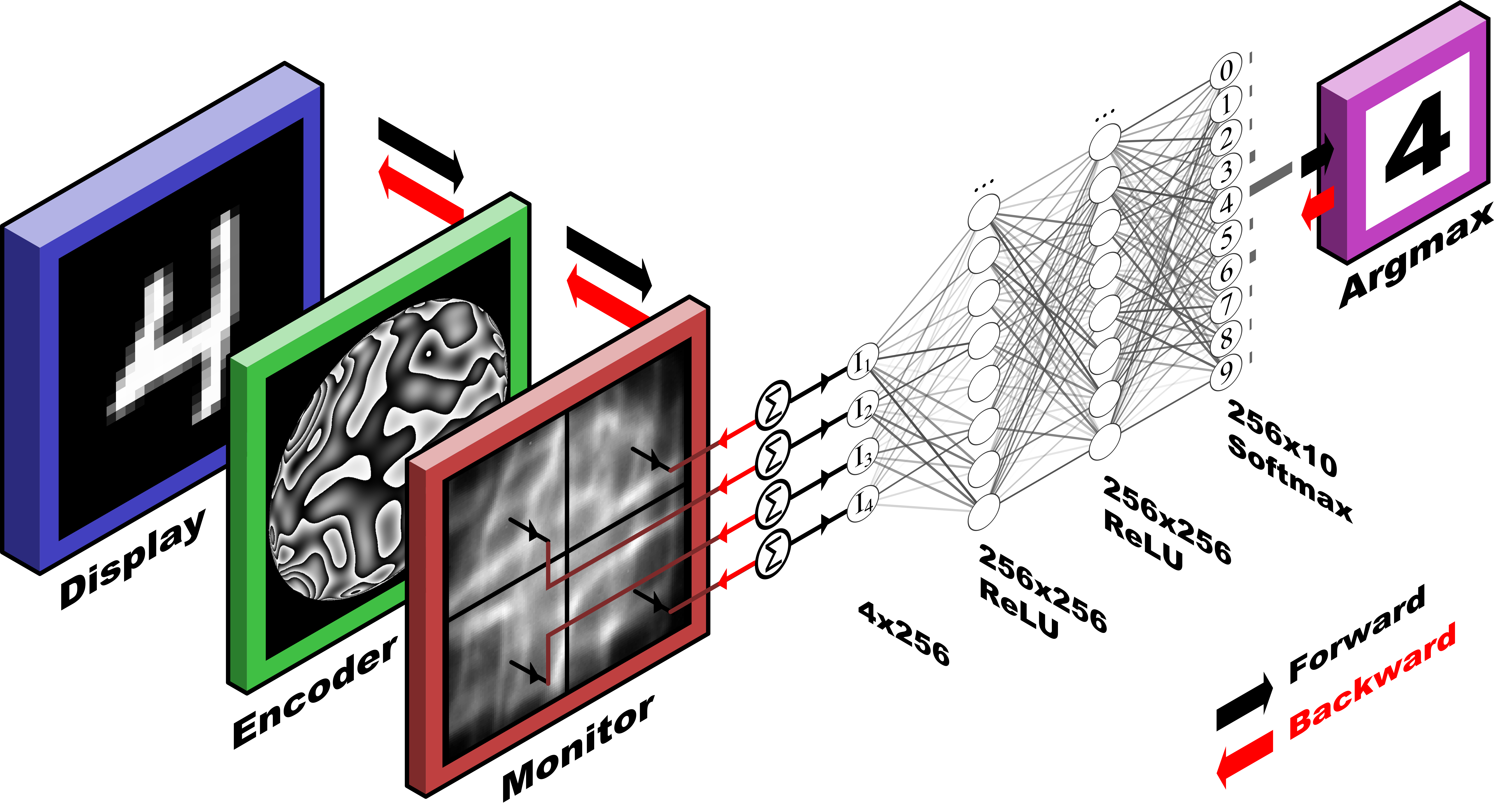
**

**Figure S1.** The hybrid optical-digital neural network is designed iteratively using an end-to-end differentiable pipeline. Each iteration consists of a forward computation of the loss, and a backward propagation of the loss. An example of the $2\times2$ encoding is shown here.

***Design of the Hybrid Optical-digital NN:***

We implemented an end-to-end differentiable pipeline to compute the gradient of the phase distribution on the meta-optical encoder with respect to the classification accuracy of the hybrid optical-digital neural network. The pipeline consists of three stages, namely, point spread function (PSF) simulation, imaging simulation, and classification. The loss function is given by the cross-entropy between the output of the hybrid neural network and the ground truth. We used TensorFlow 2.8 as the automatic differentiation engine to implement the forward computations.

In the PSF simulation stage, light with normal incidence is propagated through the meta-optics, with the phase modulation distribution denoted by $\phi(x, y)$ (“Encoder” in Fig. S1). The phase distribution is parameterized by $z_{j},$ linear combinations of Zernike polynomials $R_{n}^{m}$, in which:

$$R_{n}^{m}\left( \rho\right)=\sum_{k=0}^{\frac{n-m}{2}} \left( -1 \right)^{k}\binom{n-k}{k}\binom{n-2k}{\frac{n-m}{2}-k}\rho^{n-2k}$$

We used 200 terms of the Zernike polynomials to parameterize the phase distribution surface such that $\phi= \sum_{j=1}^{200} z_{j}R_{j}$, where $R_{j}\to R_{n}^{m}$, given $j=\frac{n\left( n+2 \right)+m}{2}$.

These polynomials are precomputed and stored into the memory, kept until the end of the entirety of the optimization loop. The Zernike polynomials are orthogonal bases that provide spatial regularization to the phase modulation of the metasurface. We found that the employment of such basis functions, instead of optimizing phase value at each spatial location, prevents the optimization from getting stuck in local minima during optimization. After computing the phase distribution, we propagate the complex field using the bandwidth-limited angular spectrum method to obtain the intensity at the focal plane, i.e., the PSF.

The second stage of the end-to-end pipeline is to convolve the PSF with the batched input MNIST images. The input images are first up-sampled using the bilinear interpolation to be the same size as the PSF intensity array. These input images are then stored in memory for the rest of the optimization routine. After that, the PSF is convolved with the input images using the Fourier convolution theorem such that $O=\mathcal{F}^{-1}\mathcal{\{F\{}PSF\mathcal{\}\cdot F\{}I\}\}$, where *O* denotes the output image, and *I* denotes the input image. The output *O* can be seen on the “Monitor” of Fig. S1. We note that this way of modelling imaging is valid only for imaging under incoherent emission, as we aim to demonstrate in this work.

The output images are then fed into a trainable digital backend ANN with sequential layers including an $N\times N$ average pooling layer, two fully connected layers with 256 units each with ReLU activation function, and finally a fully connected layer with 10 units with softmax activation function as the output layer. Note that this digital backend ANN architecture is kept the same between the hybrid optical-digital ANN as well as the purely digital ANN. Note that the $N\times N$ is equal to the input size of the digital backend. While the exact latency and power of the digital backend will depend on the technology (software, GPU or an ASIC optimized for a specific ANN), both of which are expected to monotonically increase with increasing value of $N$.

After the forward computation, we obtain the cross-entropy loss $\mathcal{L}$ between the output of the ANN and the ground truth label of the MNIST data set. The automatic differentiation algorithm then starts the backward computation in which we obtain the gradient of the loss with respect to the Zernike polynomial coefficients, namely $\partial\mathcal{L/}\partial z_{j}$. This process is visually represented in Fig. S1 by the red arrows. Then we apply this gradient to the parameters $z_{j}$ multiplied by a factor provided by the Adam optimizer with a learning rate of 0.001.

The training loop includes the forward computation of the loss function, as well as the backward computation of the loss gradient. This training loop is done iteratively until the loss converges. We find that 200 iterations are sufficient for a convergence. Both the forward and backward computations are done on an Intel Xeon @ 2.20 GHz, accelerated by a Nvidia Tesla P100 with 16GB of RAM. The optimization ran 4 times for each input size ($N\times N$).

The training and validation accuracies are displayed on Fig. S2. The training confusion matrices are shown in Fig. S3.


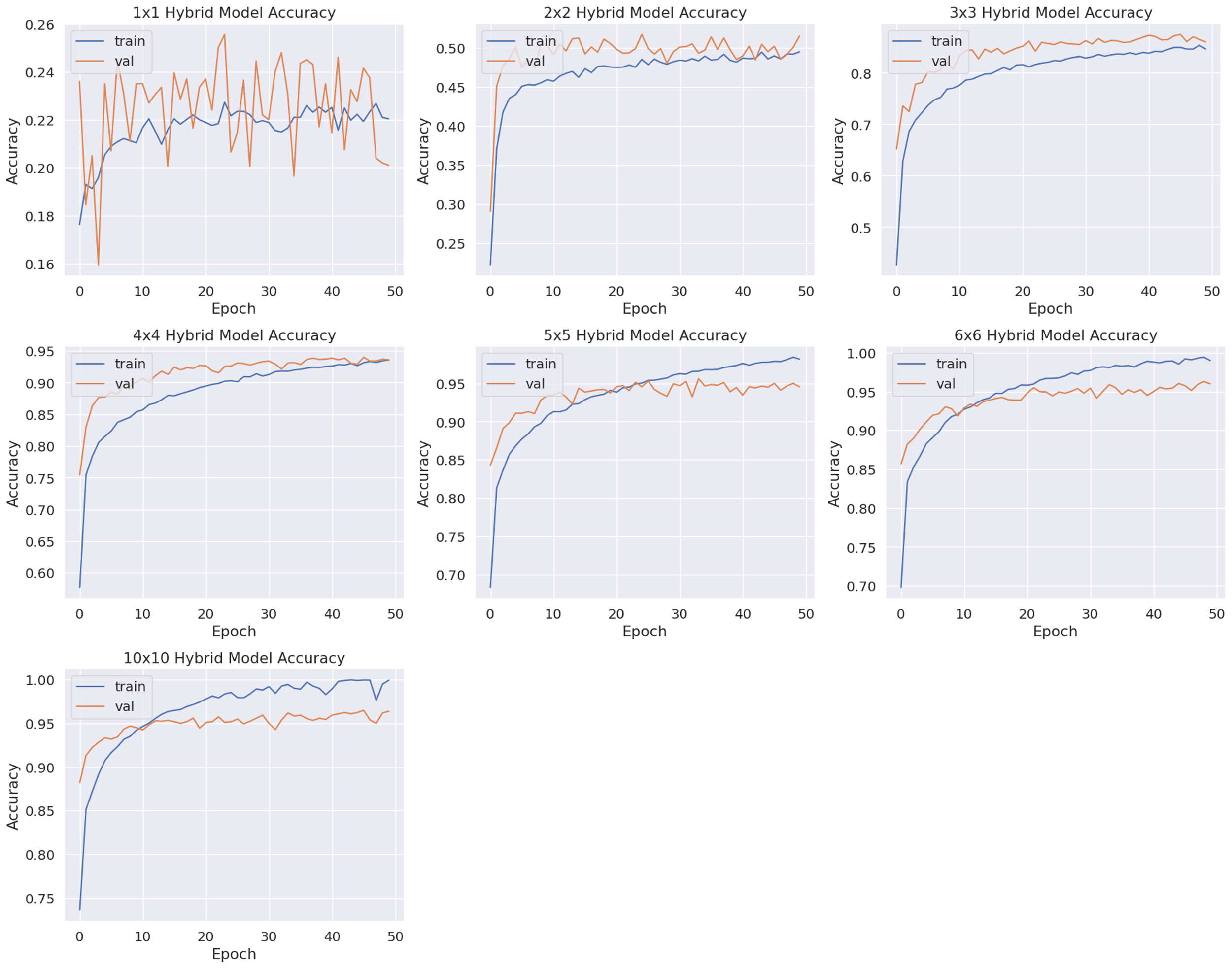


**Figure S2.** The training (train) and validation (val) accuracies of the hybrid artificial neural network classification.


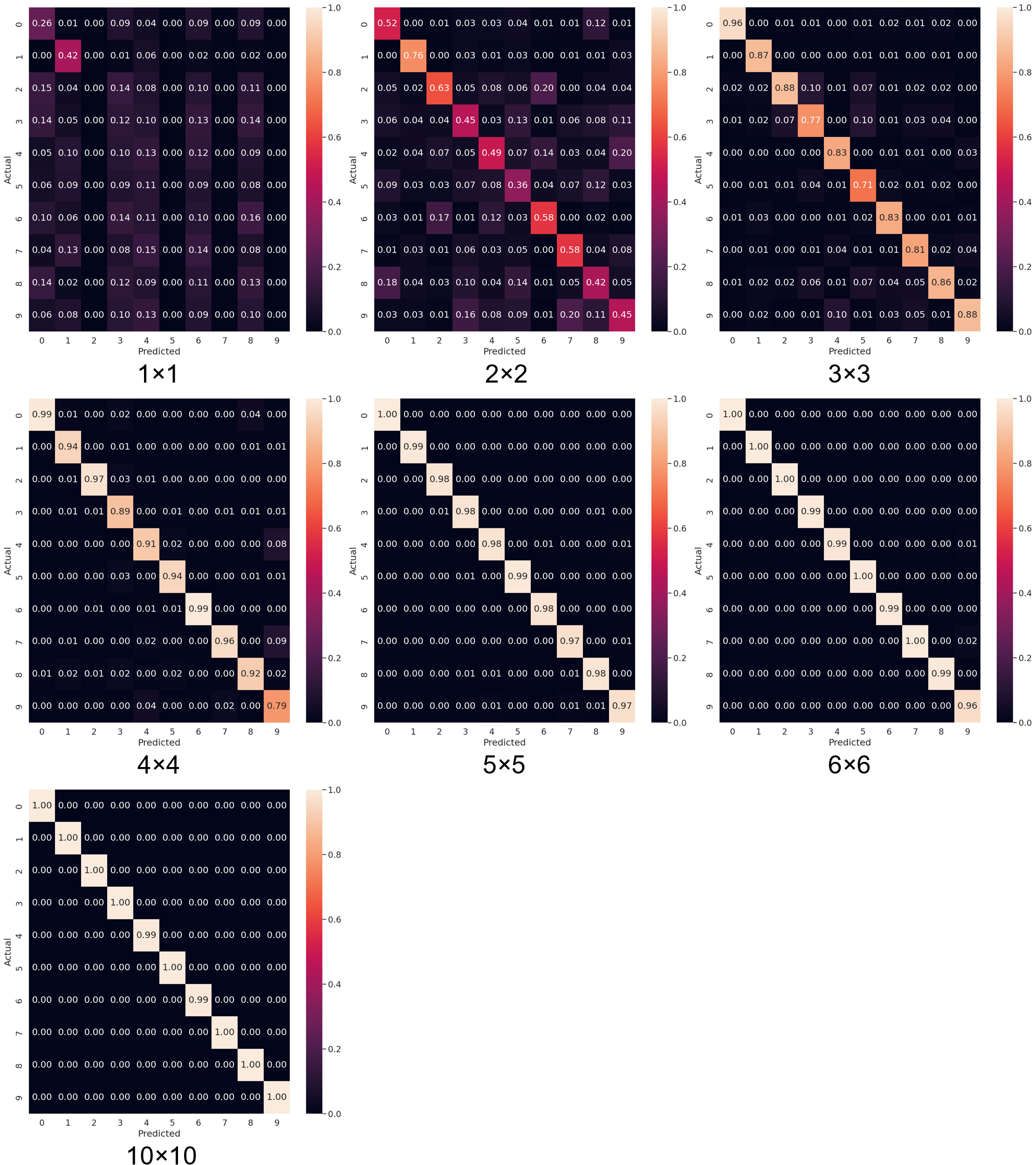


**Figure S3.** The training confusion matrices of the hybrid artificial neural networks.

**S2. Design of Purely Digital neural network**

The purely digital artificial neural network is designed with the same sequential architecture as the hybrid neural network’s digital backend, comprising four layers: an $N\times N$ average pooling layer; two layers of fully connected neurons with 256 units and ReLU activation functions; and a 10-unit fully connected layer with a softmax activation. The optimization of the neural network follows an identical routine, including the optimizer scheme and the number of iterations, and is conducted using the same hardware. The optimization routine consists of 150 epochs using the Adam optimizer with a learn rate of 0.001. Most of the optimizations converge within ~50 epochs. The training and validation accuracies are displayed on Fig. S4. The training confusion matrices are shown in Fig. S5.


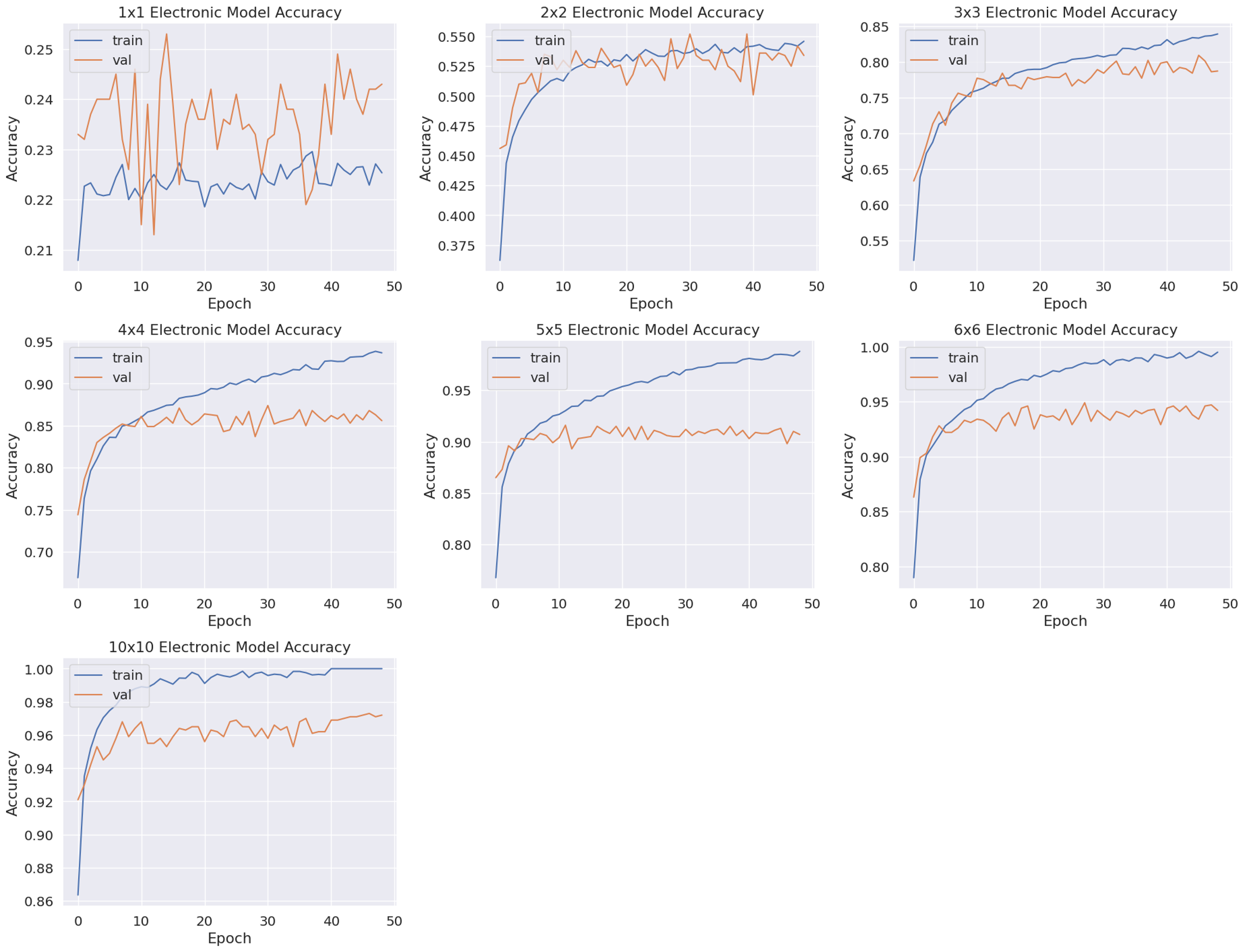


**Figure S4.** The training (train) and validation (val) accuracies of the purely electronic neural networks.

We note that, to ensure the network is well trained, we started with ~5 layers, and reduced the layers and number of neurons and trained with many different inputs. Finally, we achieved a high classification accuracy (~97%) only with two hidden layers. We emphasize that it is important to have a good training of the purely digital ANN, without which we can draw a wrong conclusion on the photonic advantage. We suspect that many of the reported optical neural network works have compared works with a poorly trained digital ANN, showing an improved classification accuracy.


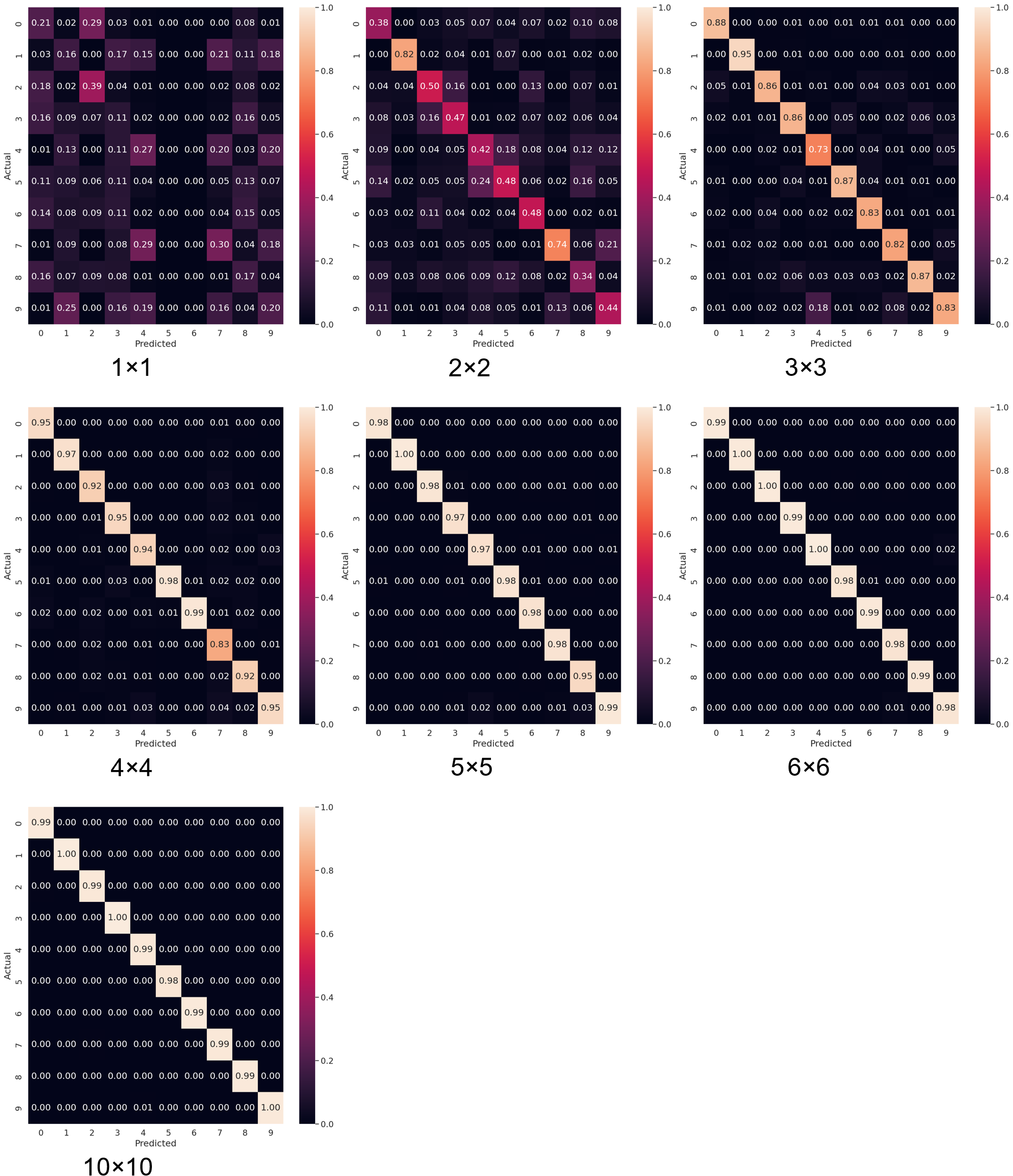


**Figure S5.** The training confusion matrices of the purely digital neural networks.

**S3. Design of the meta-optics**

The metasurface comprises a 2D array of SiN meta-atoms on a Manhattan grid, with a periodicity of 350 nm in both x and y directions. Each meta-atom is shaped as a square pillar, with a fixed height of 775 nm and lateral width parameterized to span the periodicity of the grid. This configuration is simulated using rigorous coupled-wave analysis [1], and the phase modulation of the metasurface is mapped to the corresponding meta-atom that yields the closest phase modulation to the target $\phi(x, y)$. The meta-atoms are situated on a quartz wafer with a thickness of about 500 $\mu m$.

**S4. Fabrication of the meta-optics**

For fabrication, we first deposited a ~ 775 nm thick SiN film on a 500 µm thick quartz wafer using plasma enhanced chemical vapor deposition (PECVD) in a SPTS PECVD chamber. A positive resist (ZEP 520A) was then spun onto the wafer, followed by baking at 180 °C for 3 minutes. To minimize charging effects during patterning, a conductive polymer layer (DisCharge H2O) was subsequently spun on top. The resist layer was then patterned using a 100 kV electron beam (JEOL JBX6300FS) at a dose of ~ 300 µC cm^-2^ and developed in Amyl Acetate for 2 minutes. Then a layer (~ 80 nm) of AlO_x_ was deposited using electron beam evaporation. After overnight liftoff in NMP heated at 90°C, the SiN layer was etched to the depth of 700 nm (+/-2 nm) with a remaining AlOx thickness of ~10 nm. using a mixture of C_4_F_8_/SF_6_ in an inductively coupled reactive ion etcher (Oxford PlasmaLab System 100). For SEM imaging a thin conductive Au/Pd layer was deposited to prevent charging.

**S5. Meta-optical encoder experimental details**

For the hybrid neural network experimental measurements, we first measured the label-feature pairs. The MNIST features, i.e., handwritten black and white images, are scaled up using the nearest neighbor interpolation then displayed on an OLED monitor (SmallHD 5.5 in. Focus OLED HDMI Monitor), as shown on Fig. S6. The image is first displayed via an OLED monitor, set ~10 cm away from the meta-optical encoder. The signal then goes through a custom microscope to transfer the output of the meta-optical encoder to the sensor. The power consumption of the OLED display is ~15 W. Albeit, since the sole purpose of the incoherent light source here is to provide an emulation of the real-world object, the actual power budget of the classification system should not include the OLED monitor.

**
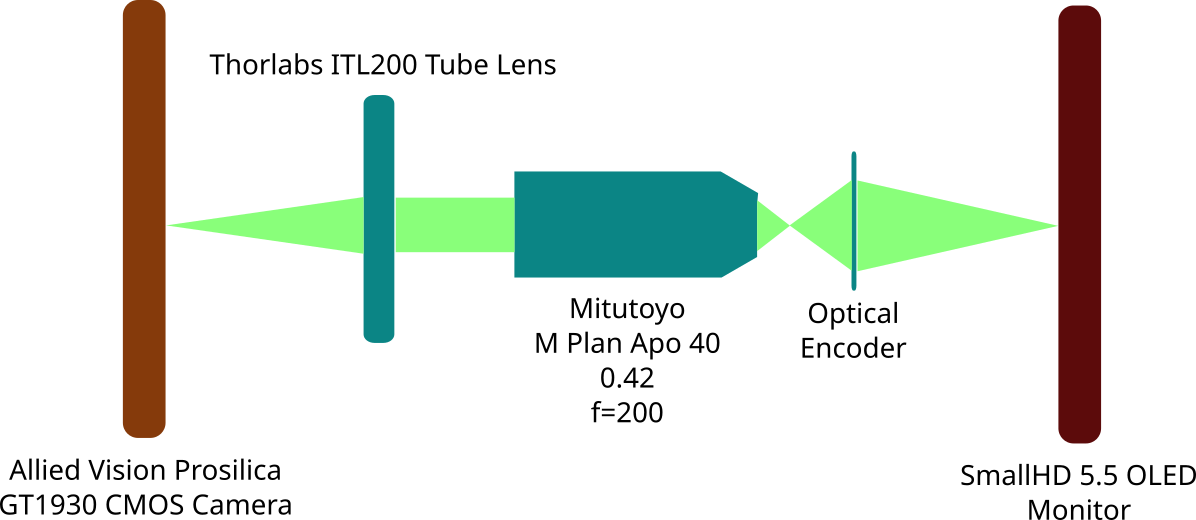
**

**Figure S6.** Schematic of the experimental setup.

Some examples of the experimental captures are shown in Fig. S8. First, the input is displayed on the OLED monitor shown on the top row. Then the encoder processes the input and projects the signal on the sensor, shown on the second row. The third row shows the encoder output after the average pooling. The fourth row shows what the purely digital ANN receives when there is no optical encoder.


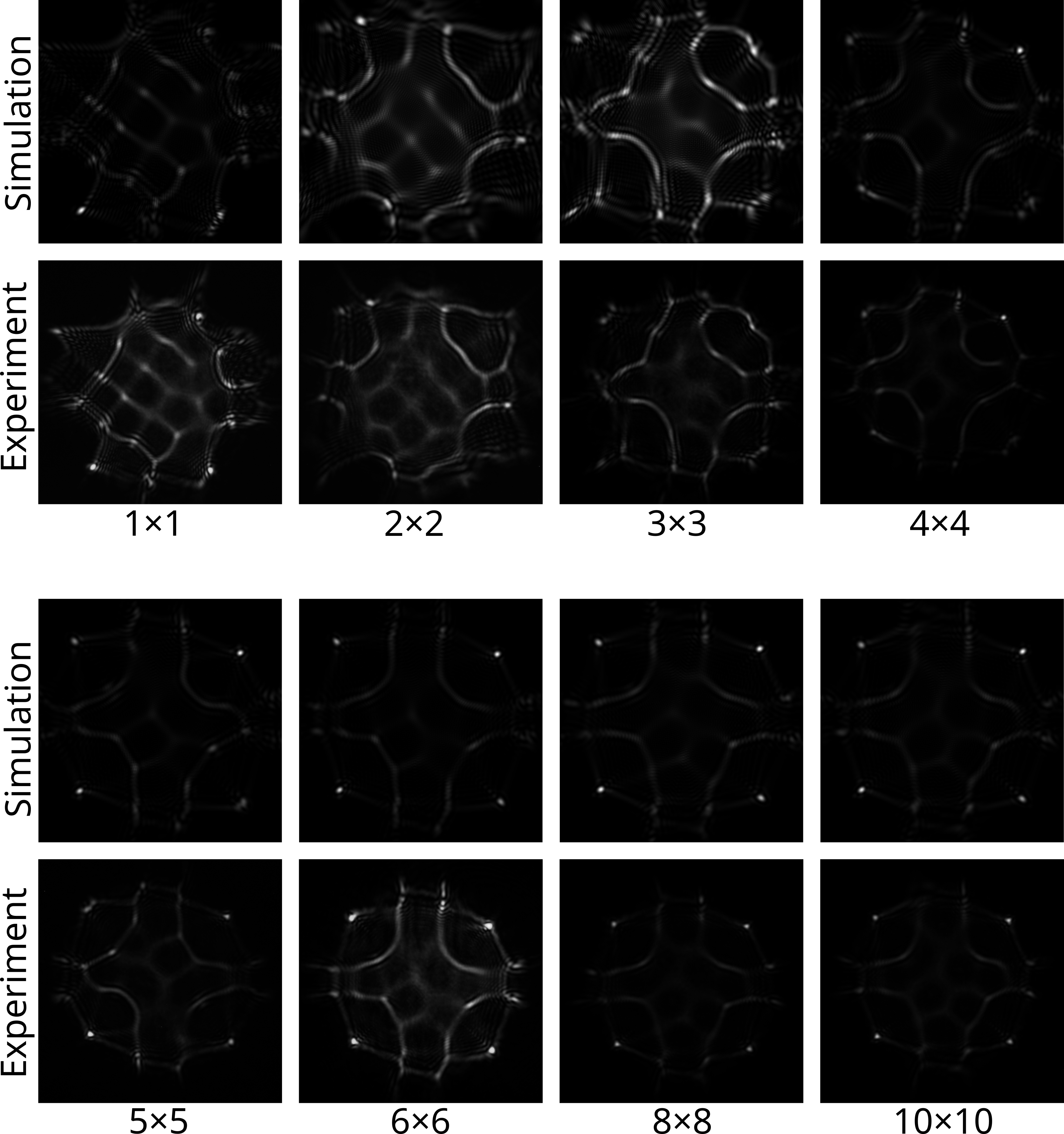


**Figure S7.** Simulation and experimental PSFs of the optical encoders.


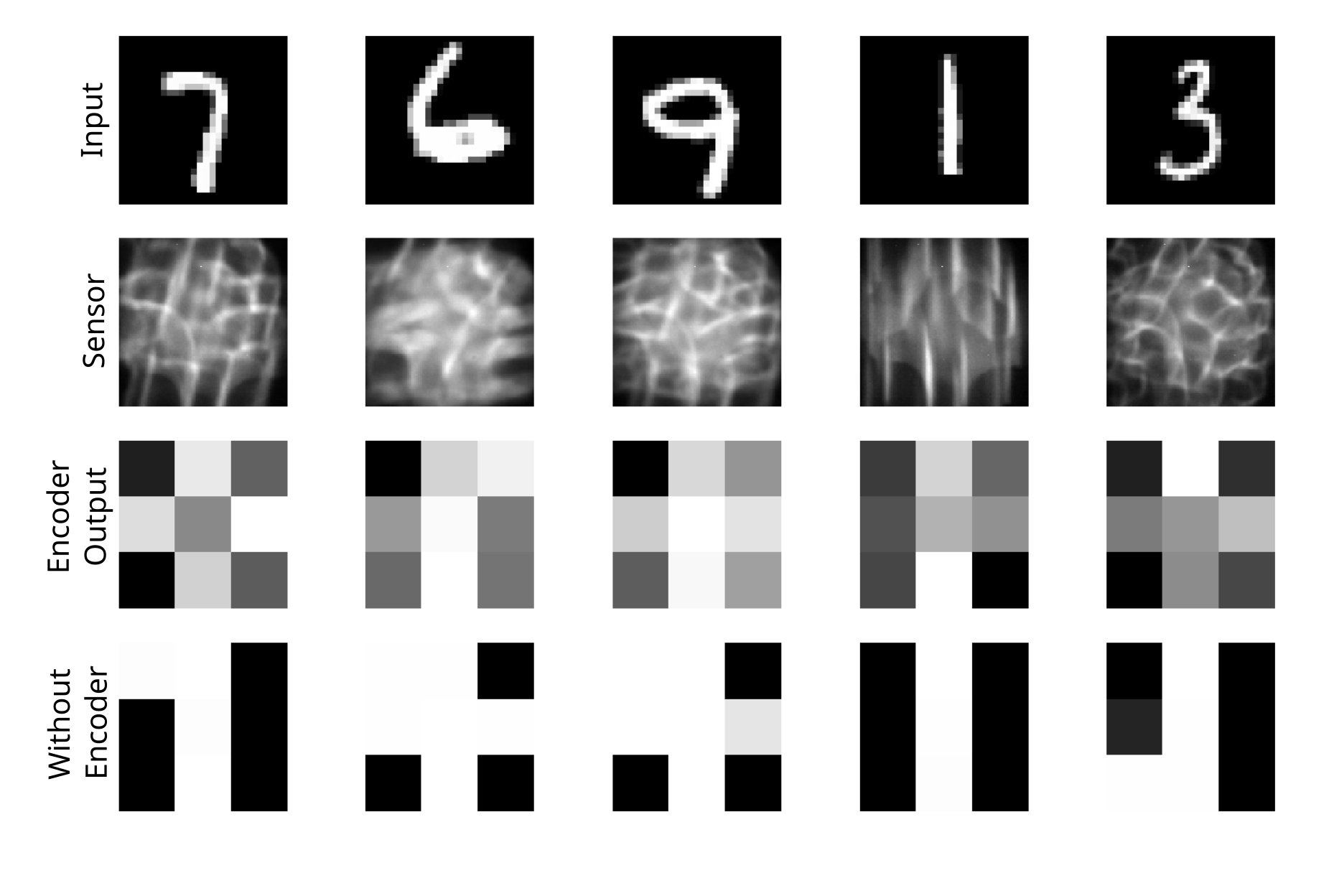


**Figure S8.** Experimental results of the displayed digits, captures, and output of the $3\times3$ encoder.

For each meta-optical encoder, we first measured the PSF responses which match up well with the simulated PSFs, seen on Fig. S7. To calibrate the image height on the OLED monitor, we displayed a cross calibration pattern with varying scaling factors and chose the closest image that resembled the simulated output. The images are then judiciously cropped to reflect the correct physical extents of the simulation. The recorded image is then decimated into an $N\times N$ image using average pooling and fed to the digital neural network. Note that one could also implement the average pooling step optically, by using a large pixel size. Such large pixel size can potentially provide benefit for low-light operations, which is important in application in mid/long wave infrared regime.

We captured 10,000 images for the training set and 2,000 images for validation. We used the captured validation data set to first calculate the accuracy of the theoretical hybrid neural network, whose accuracies are shown on Fig. 3(b). The training data set is then used to train a hardware-in-the-loop hybrid ANN with the save topology as the original hybrid ANN. This procedure is done 4 times with different random seeds during training, the validation accuracies of which are recorded. The standard deviation of the validation accuracies is also calculated and shown as the distance between the error bars shown on Fig. 3(b) in the main text.

**S6. The demonstration of benefit of optics in the existing work**

While a large body of works exists today on optics-assisted neural network, we argue that none of them showed an advantage over pure digital electronic ANN. Here, our classification of digital electronic ANN encompasses pure software solution (running on a GPU) or an application specific integrated circuit (ASIC) based accelerator. Essentially, as long as we are using CMOS-transistor based hardware, we are classifying them as digital backend. Any nano-electronic solutions, like memristors-based solutions are not included in this analysis. Based on the current works, there are largely three classes of optical neural networks, which are listed as follows.

***Integrated photonics + digital:*** There is an extensive amount of works that rely on integrated photonic based optical operations and digital backends [2]–[7]. Most of them depend on some form of arrays of switches, made of either Mach-Zehnder interferometers or ring resonators. Most of these networks are limited in terms of space-bandwidth product, which is the same as the number of waveguides. This essentially comes from the lack of dimensionality, as in integrated photonics, we effectively have one spatial dimension, making the space-bandwidth product to be $N\sim A/\lambda$, $A$ being the dimension of the chip and $\lambda$ being the optical wavelength. While the number of waveguides currently is much smaller due to technical limitations, integrated photonics is fundamentally limited by the achievable space-bandwidth product. Even with wavelength division multiplexing (WDM), the dimension of input vector remains small. As such, WDM can only provide a linear scaling of $N$. This limited space-bandwidth product necessitates time-domain multiplexing to send the data in batches, which also requires combining the data in the backend. As such most of these works did not report any excess electrical power and latency originating from the control circuit for the multiplexing. Moreover, some of the solved problems do not have an electronic benchmark and thus it is unclear if any advantage in the system level is achieved. Most of the works essentially demonstrated a similar classification accuracy against a digital ANN, but the power and latency of an application specific digital IC were not calculated.

***Free space + Digital:*** Using free-space optics (either spatial light modulator or digital micro-mirror devices), researchers can achieve much larger-space bandwidth product [8]–[14]. Thanks to the two dimensions, the space-bandwidth product scales as $N\sim\left( \frac{A}{\lambda} \right)^{2}$, $A$ being the aperture of the optics and $\lambda$ being the optical wavelength. This is a much more favorable scaling than the linear scaling in integrated photonics (even with WDM). But most of the reported works neglect the power and latency coming from the conversion between the optics and electronics. Additionally, the use of a spatial light modulator can add substantial amount of power. Comparing these power numbers with a GPU is also unfair, as the GPU is designed to be a solution for many different problems, and thus have large redundancy. For a given problem, one can optimize and design an application specific integrated circuit, with pruning/ XNOR-operations and can require much lower power.

***All-optical:*** Finally, there are a few demonstrations (both free-space and integrated photonics [3], [8], [10], [15]) of all-optical neural network, which can be implemented without a digital backend. Some of them are fully linear (without any nonlinearity) and as such they cannot be used for complicated datasets. Moreover, even there, multiple layers of optics are generally used, each with additional scattering losses. Thus, the input power might be high, and they also require a laser to encode the electronic information. It is unclear what kind of wall-plug efficiency has been achieved in these cases. Thus, the true power is not reported. Others employed nonlinearity either based on saturable absorption, electromagnetic induced transparency in cold atoms, or optoelectronic nonlinearity (image intensifier or optically induced electro-optic nonlinearity). While some of them did demonstrate operation with MNIST data set, the power consumption of all the possible sources is not accounted for, for example, no laser powers are accounted for to cool down the atom. As such, a true estimation of system level power/ latency is missing in all these works, and thus the claims of benefit over purely digital ANN were not established.

**References**

[1] V. Liu and S. Fan, “S4 : A free electromagnetic solver for layered periodic structures,” *Comput. Phys. Commun.*, vol. 183, no. 10, pp. 2233–2244, Oct. 2012, doi: 10.1016/j.cpc.2012.04.026.

[2] X. Xu *et al.*, “11 TOPS photonic convolutional accelerator for optical neural networks,” *Nature*, vol. 589, no. 7840, pp. 44–51, Jan. 2021, doi: 10.1038/s41586-020-03063-0.

[3] F. Ashtiani, A. J. Geers, and F. Aflatouni, “An on-chip photonic deep neural network for image classification,” *Nature*, vol. 606, no. 7914, pp. 501–506, Jun. 2022, doi: 10.1038/s41586-022-04714-0.

[4] Y. Shen *et al.*, “Deep learning with coherent nanophotonic circuits,” *Nat. Photonics*, vol. 11, no. 7, pp. 441–446, Jul. 2017, doi: 10.1038/nphoton.2017.93.

[5] A. Sludds *et al.*, “Delocalized photonic deep learning on the internet’s edge,” *Science*, vol. 378, no. 6617, pp. 270–276, Oct. 2022, doi: 10.1126/science.abq8271.

[6] J. Feldmann *et al.*, “Parallel convolutional processing using an integrated photonic tensor core,” *Nature*, vol. 589, no. 7840, pp. 52–58, Jan. 2021, doi: 10.1038/s41586-020-03070-1.

[7] S. Bandyopadhyay *et al.*, “Single chip photonic deep neural network with accelerated training.” arXiv, Aug. 02, 2022. Accessed: Apr. 04, 2023. [Online]. Available: http://arxiv.org/abs/2208.01623

[8] A. Ryou *et al.*, “Free-space optical neural network based on thermal atomic nonlinearity,” *Photonics Res.*, vol. 9, no. 4, p. B128, Apr. 2021, doi: 10.1364/PRJ.415964.

[9] T. Wang, S.-Y. Ma, L. G. Wright, T. Onodera, B. C. Richard, and P. L. McMahon, “An optical neural network using less than 1 photon per multiplication,” *Nat. Commun.*, vol. 13, no. 1, p. 123, Jan. 2022, doi: 10.1038/s41467-021-27774-8.

[10] T. Wang *et al.*, “Image sensing with multilayer nonlinear optical neural networks,” *Nat. Photonics*, Mar. 2023, doi: 10.1038/s41566-023-01170-8.

[11] J. Chang, V. Sitzmann, X. Dun, W. Heidrich, and G. Wetzstein, “Hybrid optical-electronic convolutional neural networks with optimized diffractive optics for image classification,” *Sci. Rep.*, vol. 8, no. 1, p. 12324, Aug. 2018, doi: 10.1038/s41598-018-30619-y.

[12] J. Spall, X. Guo, and A. I. Lvovsky, “Hybrid training of optical neural networks,” *Optica*, vol. 9, no. 7, p. 803, Jul. 2022, doi: 10.1364/OPTICA.456108.

[13] D. Mengu, Y. Luo, Y. Rivenson, and A. Ozcan, “Analysis of Diffractive Optical Neural Networks and Their Integration With Electronic Neural Networks,” *IEEE J. Sel. Top. Quantum Electron.*, vol. 26, no. 1, pp. 1–14, Jan. 2020, doi: 10.1109/JSTQE.2019.2921376.

[14] H. Zhang *et al.*, “An optical neural chip for implementing complex-valued neural network,” *Nat. Commun.*, vol. 12, no. 1, p. 457, Jan. 2021, doi: 10.1038/s41467-020-20719-7.

[15] X. Lin *et al.*, “All-optical machine learning using diffractive deep neural networks,” *Science*, vol. 361, no. 6406, pp. 1004–1008, Sep. 2018, doi: 10.1126/science.aat8084.
